# Role of the voltage window on the capacity retention of P2-Na_2/3_[Fe_1/2_Mn_1/2_]O_2_ cathode material for rechargeable sodium-ion batteries

**DOI:** 10.1038/s42004-022-00628-0

**Published:** 2022-02-01

**Authors:** Maider Zarrabeitia, Francesco Nobili, Oier Lakuntza, Javier Carrasco, Teófilo Rojo, Montse Casas-Cabanas, Miguel Ángel Muñoz-Márquez

**Affiliations:** 1 Centre for Cooperative Research on Alternative Energies (CIC energiGUNE), Basque Research and Technology Alliance (BRTA), Alava Technology Park, Albert Einstein 48, 01510 Vitoria-Gasteiz, Spain; 2 School of Science and Technology—Chemistry Division, University of Camerino, Via Madonna delle Carceri, 62032 Camerino, Italy; 3 Departamento de Química Inorgánica, Universidad del País Vasco UPV/EHU, P.O. Box 664, 48080 Leioa, Spain; 4 IKERBASQUE, Basque Foundation for Science, María Díaz de Haro 3, 48013 Bilbao, Spain; 5 Present Address: Helmholtz Institute Ulm (HIU), Helmholtzstrasse 11, 89081 Ulm, Germany; 6 Present Address: Karlsruhe Institute of Technology (KIT), P.O. Box 3640, 76021 Karlsruhe, Germany; 7 Present Address: School of Science and Technology—Chemistry Division, University of Camerino, Via Madonna delle Carceri, 62032 Camerino, Italy

**Keywords:** Batteries, Batteries

## Abstract

P2-Na_2/3_[Fe_1/2_Mn_1/2_]O_2_ layered oxide is a promising high energy density cathode material for sodium-ion batteries. However, one of its drawbacks is the poor long-term stability in the operating voltage window of 1.5–4.25 V vs Na^+^/Na that prevents its commercialization. In this work, additional light is shed on the origin of capacity fading, which has been analyzed using a combination of experimental techniques and theoretical methods. Electrochemical impedance spectroscopy has been performed on P2-Na_2/3_[Fe_1/2_Mn_1/2_]O_2_ half-cells operating in two different working voltage windows, one allowing and one preventing the high voltage phase transition occurring in P2-Na_2/3_[Fe_1/2_Mn_1/2_]O_2_ above 4.0 V vs Na^+^/Na; so as to unveil the transport properties at different states of charge and correlate them with the existing phases in P2-Na_2/3_[Fe_1/2_Mn_1/2_]O_2_. Supporting X-ray photoelectron spectroscopy experiments to elucidate the surface properties along with theoretical calculations have concluded that the formed electrode-electrolyte interphase is very thin and stable, mainly composed by inorganic species, and reveal that the structural phase transition at high voltage from P2- to “Z”/OP4-oxygen stacking is associated with a drastic increased in the bulk electronic resistance of P2-Na_2/3_[Fe_1/2_Mn_1/2_]O_2_ electrodes which is one of the causes of the observed capacity fading.

## Abstract (web summary)

P2-Na_2/3_[Fe_1/2_Mn_1/2_]O_2_ is a promising high energy density cathode material for rechargeable sodium-ion batteries, but its poor long-term stability in the operating voltage window of 1.5–4.25 V vs Na^+^/Na hinders its commercial application. Here, the authors use a combination of electrochemical impedance spectroscopy, X-ray photoelectron spectroscopy, and DFT calculations to investigate the origin of the capacity fading, which is attributed to an increase in bulk electronic resistance at high voltage that, among other factors, is nested in a structural phase transition.

## Introduction

Reversible extraction/insertion of Na^+^ into host structures was already demonstrated in the 1980s with layered oxides^1,2^. Nowadays, pushed by the increasing need for more efficient low-cost energy storage devices, sodium-ion batteries (SIBs) are becoming an alternative for large-scale applications and light electromobility^3–5^. This is nested in the fact that sodium precursors are evenly distributed in the Earth’s crust and are cheaper and more abundant than lithium ones^6–8^. Due to their similar chemical properties, many lithium-based analog electrode materials have been proposed as cathodes for SIBs^5^. Therefore, a wide variety of sodium-based cathode materials have been studied, including polyanionic materials (phosphates, pyrophosphates, and mixed polyanions), organic compounds, Prussian Blue analogs (PBAs), and layered oxides^9–14^. Each one of these materials has advantages and limitations, for example, polyanionic materials exhibit very good capacity retention due to their stable 3D framework, in contrast, their specific capacity is typically lower than many layered oxides^9,10^; organic compounds show massive capacity fading due to their dissolution in the organic carbonate-based electrolyte^11^; PBAs usually deliver lower capacities at low operating voltage than layered oxides^12^. Layered oxides with general formula Na_*x*_TMO_2_ (TM = transition metal/s such as Co, Mn, Fe, Ni, Ti, V, etc., as well as alkali metals namely Li, K, and Mg) can deliver high specific capacity, but their cycle life should be even greater enhanced^13–15^. Among all layered oxides, P2-Na_2/3_[Fe_1/2_Mn_1/2_]O_2_ is one of the most promising cathode materials in terms of cost-efficiency and energy density^16^. It is made from Earth abundant elements and delivers a high reversible capacity of 190 mAh g^−1^ when it is cycled in the voltage range of 1.5–4.3 V vs Na^+^/Na using metallic sodium as the counter electrode. Moreover, in a full-cell configuration, using hard carbon (HC) as an anode, P2-Na_2/3_[Fe_1/2_Mn_1/2_]O_2_ delivers a reversible capacity of 185 mAh g^−1^ with an average cell voltage of 2.75 V^17^; which is comparable to the prototype cells developed by Faradion using a quaternary layered oxide (Na_a_Ni_(1−*x*−*y*−*z*)_Mn_*x*_Mg_*y*_Ti_*z*_O_2_) as cathode and HC as anode^18^. Albeit the good energy density values obtained from P2-Na_2/3_[Fe_1/2_Mn_1/2_]O_2_ layered oxide, capacity retention is still one of its major weaknesses and it has been related to phase transitions involving different stacking sequences when the sodium concentration changes while inducing large volume changes and exfoliation of the layered oxide at particle surface^19^.

The structural evolution of P2-Na_2/3_[Fe_1/2_Mn_1/2_]O_2_ (*P*6_3_/*mmc*) occurs *via* a solid-solution mechanism in the voltage range of 2.0–4.0 V vs Na^+^/Na (from 0.67 to 0.36 Na^+^ *per formula* (*p.f*.) unit)^15,20^. The repulsion between [TMO_2_] slabs, induced by Na^+^ extraction from the interlayer space, leads to a decrease of *a* parameter while *c* parameter increases. Above 4.0 V (Na^+^ *p.f*. unit = 0.36) and up to 4.1 V vs Na^+^/Na (Na^+^ *p.f*. unit = 0.26) a second phase appears showing a biphasic region. Above 4.1 V, the newly formed phase propagates as solid-solution until the end of the Na^+^ extraction process (4.1–4.3 V vs Na^+^/Na; Na^+^ *p.f*. unit = 0.19). The exact structure of this second phase at high voltage is still controversial due to the fact that it is formed by layer gliding and therefore structural disorder increases. Indeed, this phase could not be clearly indexed by powder X-ray diffraction (XRD) measurements and it is mainly referred as “Z” phase, OP4-type structure (scape group (S.G): *P*-6*m*2 or *P*6_3_) or P–O intergrowth phase^16,20–24^, which consists of alternating layers with P- and O-interlayer sites that result from the gliding of half [TMO_2_] layers as determined by synchrotron XRD^16^. During Na^+^ insertion, the reverse reaction mechanism is observed: starting from “Z”/OP4 solid-solution region, followed first by a biphasic region until 3.10 V vs Na^+^/Na (Na^+^ *p.f*. unit = 0.44) and later by a P2 solid-solution region. Finally, below 2.0 V vs Na^+^/Na (Na^+^ *p.f*. unit = 0.8), the P2-phase coexists with a new distorted P’2-phase (S.G: *Cmcm*)^20^. In addition, the described structural mechanism of P2-Na_2/3_[Fe_1/2_Mn_1/2_]O_2_ is not affected by the electrolyte used^16,20–22^. On the other hand, ex-situ and in-situ XRD show that all the structural transformations are reversible^22^, although a slight broadening of the peaks and the gradual intensity decrease in the second cycle suggest that the crystallinity of the material is reduced upon electrochemical cycling^21^. Indeed, ex-situ electronic microscopy analysis —scanning electronic microscopy (SEM) and transmission electronic microscopy (TEM)— clearly shows the exfoliation of the P2-type layered oxides after cycling, which has been attributed as one of the causes of the poor capacity retention of P2-Na_2/3_[Fe_1/2_Mn_1/2_]O_2_^25,26^. The structural transformations are known to be detrimental to the reversible capacity —mainly due to the huge volume changes involved—^24^, although the exact mechanism and effect on the physical properties of such impoverishment are not understood yet.

Several strategies have been developed in order to improve its long-term stability. One of the most successful approaches is to partially substitute the TM by an electrochemically active or inactive element/s (such as Ni, Co, Cu, Mg, Al, Ti, K, Li, and so on) giving rise to ternary or quaternary compounds which, despite typically displaying lower capacity values, exhibit better capacity retention and in some cases higher operating voltage^27–34^. The origin of such enhanced electrochemical performance is still unclear and has been attributed to different factors, such as improvement of the structural stability, reduction of the volume change between Na^+^extracted and Na^+^inserted states, increase of the sodium interlayer distance, buffering of the Jahn-Teller induced distortion on Mn(III), and or controlling the distribution of Na^+^^32,34–38^. Interestingly, the (slightly) doped P2-layered oxides that exhibit improved cycling stability, do not show a high voltage phase transition or the volume changes between P2 and “Z”/OP4 significantly reduced^19,30,33,34,39,40^. This observation suggested that the high voltage phase formation should be avoided. Consequently, an alternative approach to improve the capacity retention has been studied -not only for P2-Na_2/3_[Fe_1/2_Mn_1/2_]O_2_ but also for other P2-layered oxides— which is the reduction of the operating voltage window, from >4.3 to 4.0 V vs Na^+^/Na, avoiding the phase transition that has been observed upon cycling at high voltage, although at the cost of a lower capacity as well^21,23,24,41,42^. The exact impact of reducing the operating voltage window on the electrochemical performance of layered oxides has been attributed to the improvement of the structural stability upon electrochemical cycling, however, further studies should be carried out to clearly discern the role of the operating voltage window on the electrochemical, physical, and structural properties of P2-layered oxides.

In this work, P2-Na_2/3_[Fe_1/2_Mn_1/2_]O_2_ cathode material is studied by means of electrochemical impedance spectroscopy (EIS) in two operating voltage windows. On the one hand, from 1.5 to 4.25 V (P2-NFMO-LV), where the P2- to “*Z*”/OP4-type phase transition will occur. On the other hand, from 2.0 to 4.0 V (P2-NFMO-SV), avoiding the above-mentioned phase transition. These studies are completed with compositional studies of the electrode-electrolyte interface by means of X-ray photoelectron spectroscopy (XPS) and with density functional theory (DFT) simulations of the electronic structure. The results show that the phase transition occurring at high voltage has a profound impact on the electronic and ionic transport properties of P2-Na_2/3_[Fe_1/2_Mn_1/2_]O_2_ electrode, and such changes directly impact in capacity retention. The obtained  results in this investigation can be extrapolated to other P2-layered oxides with different TM compositions and will help clarify the role of the operating voltage window and high voltage phase formation in the electrochemical properties of the P2-layered oxides.

## Results and discussion

### P2-Na_2/3_[Fe_1/2_Mn_1/2_]O_2_ cycled in the 1.5–4.25 V vs Na^+^/Na voltage window: P2-NFMO-LV electrode

**Fig. 1:**
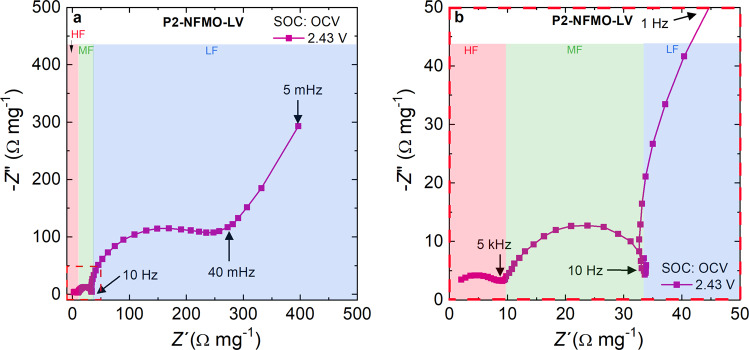
Nyquist plot of P2-NFMO-LV electrode at OCV (2.43 V vs Na^+^/Na). The impedance data from **a** 100 kHz to 5 mHz and **b** 100 kHz to 1 Hz. The frequency regions are highlighted (HF-red, MF-green, and LF-blue).

For the first voltage window studied, the Nyquist plot of the impedance dispersion recorded at an open circuit voltage (OCV—2.43 V vs Na^+^/Na) is shown in Fig. 1a. Three semicircles are observed at different frequencies: low-frequency (LF) below 10 Hz, medium-frequency (MF) in the 5 kHz–10 Hz range and, high-frequency (HF) above 5 kHz. The latter one is better observed once the impedance dispersion is enlarged (see Fig. 1b). The HF semicircle corresponds to the Na^+^ migration resistance through the electrode-electrolyte interphase (EEI) and is labeled as *R*_EEI_. The oxidation of the electrolyte and subsequent formation of the EEI is not expected at OCV, but a similar EEI is chemically formed before cycling, as confirmed by XPS and in agreement with previous EEI studies of SIB electrodes^43–46^. The high reactivity upon air/moisture exposure of the layered oxide^47^, the dehydrofluorination reaction of polivinylidene fluoride (PVdF), which takes place during electrode preparation as confirmed by solid-state nuclear magnetic resonance^43^, and the use of metallic sodium as counter electrode contribute^48–50^ to form a passivation surface layer composed by carbonate species originated by electrolyte decomposition reactions. The MF semicircle corresponds to charge-transfer resistance (*R*_CT_) and accumulation of charge in the interfacial double layer (*C*_DL_), while the LF region process can be correlated to the bulk electronic conductivity, as already described for other layered oxides electrode materials among others^51–54^. Additionally, in the very LF region (40–5 mHz) a sloping line at ~45° with respect to the real axis (*Z*´) can be observed which corresponds to the Na^+^ solid-state diffusion.

**Fig. 2:**
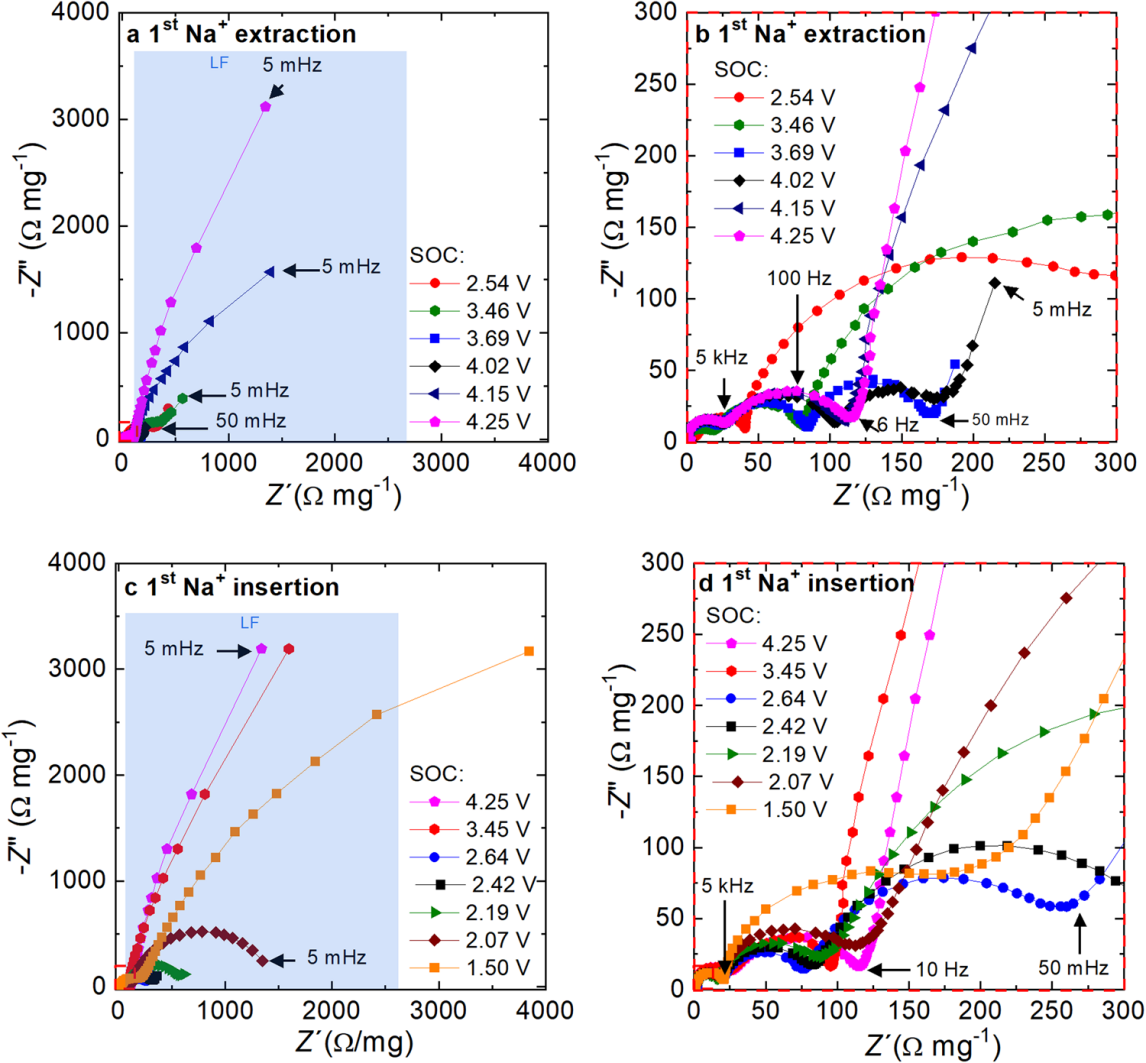
Nyquist plots of P2-NFMO-LV electrode upon the first cycle. In the top panels, during first Na^+^ extraction: at 2.53 V (red point), 3.46 V (green hexagon), 3.69 V (blue square), 4.02 V (black rhombus), 4.15 V (navy triangle), and 4.25 V (pink pentagon) in the frequency range of **a** 100 kHz to 5 mHz and **b** 100 kHz to 1 Hz/50 mHz which is a zoom-in of the very low real impedance component. In the bottom panels, during first Na^+^ insertion: at 4.25 V (pink pentagon), 3.45 V (red hexagon), 2.64 V (blue point), 2.42 V (black square), 2.19 V (green triangle), 2.07 V (brown rhombus), and 1.50 V (orange square) in the frequency range of **c** 100 kHz to 5 mHz and **d** 100 kHz to 1 Hz/50 mHz which is a zoom-in of the very low real impedance component. The LF region is highlighted in blue.

**Fig. 3:**
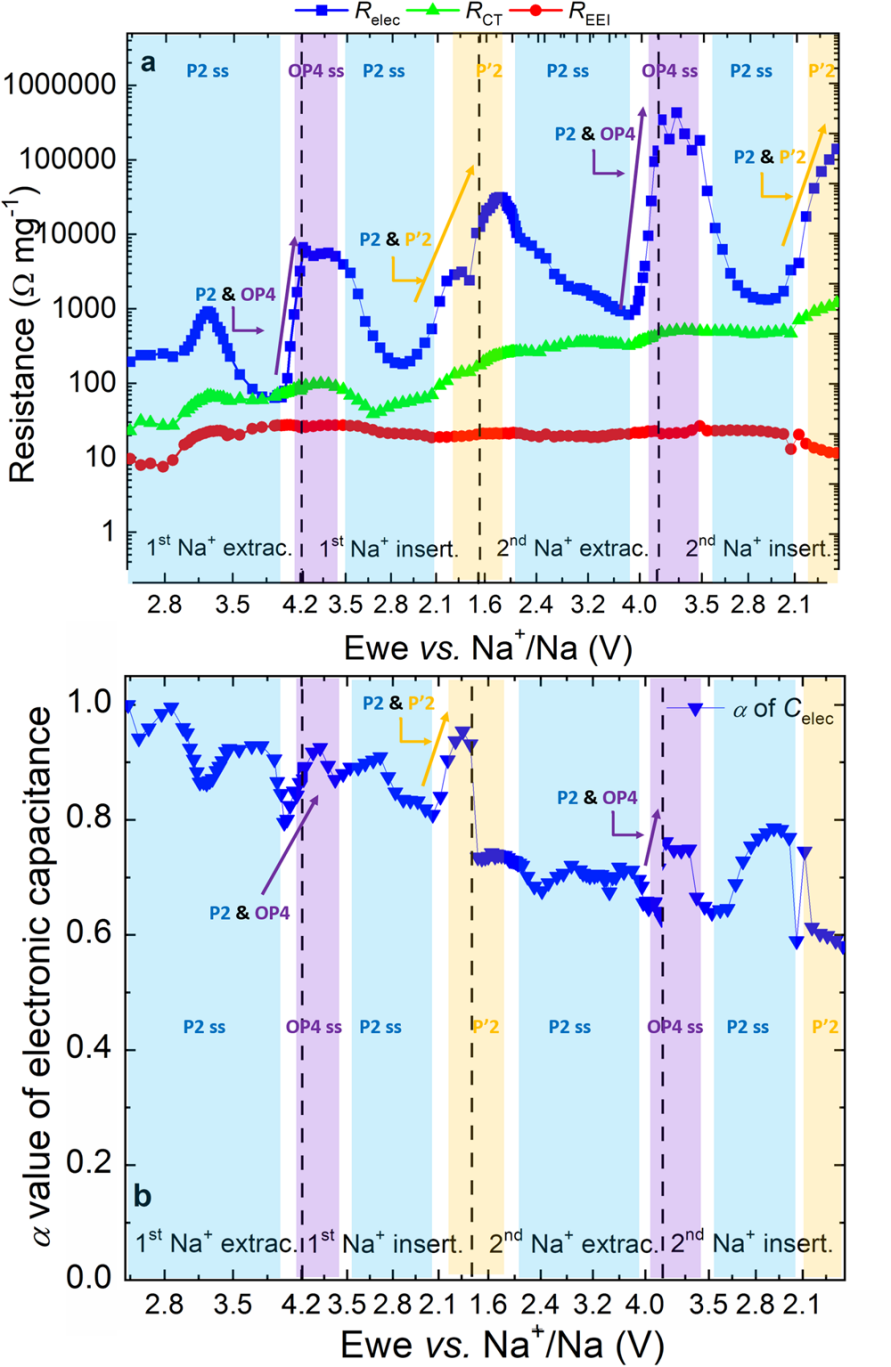
Fitted resistance values for the first two electrochemical cycles of the P2-NFMO-LV electrode. **a** Resistance trend of *R*_EEI_ (red point), *R*_CT_ (green triangle), *R*_elec_ (blue square) and **b** *α* factor of the CPE associated with *C*_elec_ (blue inverted triangle). The structural evolution highlighted as colored regions and labeled upon electrochemical cycling is included in the figure for guidance^15, 20^.

The EIS spectrum of the P2-NFMO-LV electrode were collected every 45 mV during the first two cycles in the voltage range of 1.5–4.25 V vs Na^+^/Na and the Nyquist plots shown in Fig. 2 correspond to some relevant potential values of the first Na^+^ extraction (Fig. 2a, b) and insertion (Fig. 2c, d) processes. The overall trend of the resistance values obtained from the fit to the equivalent circuit detailed in the experimental section of all impedance spectra (133 in total) measured during the first two cycles is shown in Fig. 3.

The described equivalent circuit (more details in the Methods section) is a modification of the surface model proposed by Aurbach and co-workers^55,56^. This model, developed for graphite electrodes, assumes that the active material has good electronic conductivity, but it is known that layered oxides are poor electronic conductors. Therefore, in the used equivalent circuit model, the poor electronic conductivity of the P2-layered oxides has been considered as an extra resistance and capacitance labeled as bulk electronic resistance (*R*_elec_) and capacitance arising from charge accumulation (*C*_elec_).

Figures 2 and 3 reveal that the main change in the Nyquist plots is observed in the LF semicircle (blue region of Fig. 2a, c), indicating a variation of the *R*_elec_ (blue square of Fig. 3) upon Na^+^ extraction/insertion which can be related to modifications in the crystalline structure. Similar changes in the EIS behavior at LFs have been observed in other layered oxide cathodes and anodes^53,54,57–60^.

After comparing the first with the second cycle, it is found that *R*_elec_ (blue square of Fig. 3) continuously increases although several oscillations take place depending on the voltage and Na^+^ content, which are related to the structural evolution of P2-Na_2/3_[Fe_1/2_Mn_1/2_]O_2_. In the P2 solid-solution region (blue region of Fig. 3), *R*_elec_ decreases upon Na^+^ extraction while increases upon Na^+^ insertion after raising the minimum *R*_elec_ values due to the OP4 effect. Except for the first Na^+^ extraction in the 3.00–3.33 V vs Na^+^/Na range, probably due to the fact that the electron transfer is easier in mixed valence states (Fe^3+^/Fe^4+^ couple —3.5 V vs Na^+^/Na) than when only Mn^4+^ and Fe^3+^ are present on the P2-Na_2/3_[Fe_1/2_Mn_1/2_]O_2_^21^. This means that in the P2 solid-solution region, the P2-NFMO-LV electrode exhibits a reversible behavior in terms of electronic conductivity, becoming a better electronic conductor during Na^+^ extraction. Nevertheless, the *R*_elec_ trend is interrupted when the P2-phase is transformed into “*Z* ”/OP4-type structure, above 4.0 V vs Na^+^/Na during Na^+^ extraction (violet region of Fig. 3), and when the distorted P’2 phase is observed, below 2.0 V vs Na^+^/Na during Na^+^ insertion (yellow region of Fig. 3). These increments of *R*_elec_ at 4.0 and 2.0 V vs Na^+^/Na progressively reduce the bulk electronic conductivity of P2-NFMO-LV electrode upon electrochemical cycling, as indicated by the overall increase of *R*_elec_, where the increment of *R*_elec_ at the second cycle is even higher than in the first one. This massive *R*_elec_ increase suggests that the large volume change occurring during these phase transitions —mainly from P2 to “Z”/OP4— result in electrical isolation (loss of contact) among the active material particles, as well as the conductive carbon, and current collector.

In the MF semicircle of the Nyquist plot, *R*_CT_ (green triangle of Fig. 3) constantly increases overall upon electrochemical cycling. This is expected since the phase transitions upon electrochemical cycling induce the irreversible formation of grain boundaries, a mosaic texture, exfoliation of the layer or/and increase of interfacial microstrains, which was attributed as one of the causes of the capacity fading of P2-layered oxides, including P2-Na_2/3_[Fe_1/2_Mn_1/2_]O_2_^25,26,61^.

In parallel, *R*_EEI_ (red points of Fig. 3) constantly increases upon first Na^+^ extraction, while above 3.00 V vs Na^+^/Na it remains constant. This behavior suggests that the formed EEI is overall stable during the electrochemical cycling. Nonetheless, there is a small drop in the second  Na^+^ insertion, more previously in the P′2 region (below 2.1 V vs Na^+^/Na). There are two main factors that can cause the lower *R*_EEI_. On the one hand, some electrical contact problem —note that there is a glitch at 2.4 V, while *R*_CT_ also suffers the drop. On the other hand, some slight modifications to the EEI. However, the stability of local regions of the interphase, *i.e.*, the outermost surface region, cannot be corroborated by means of EIS.

**Fig. 4:**
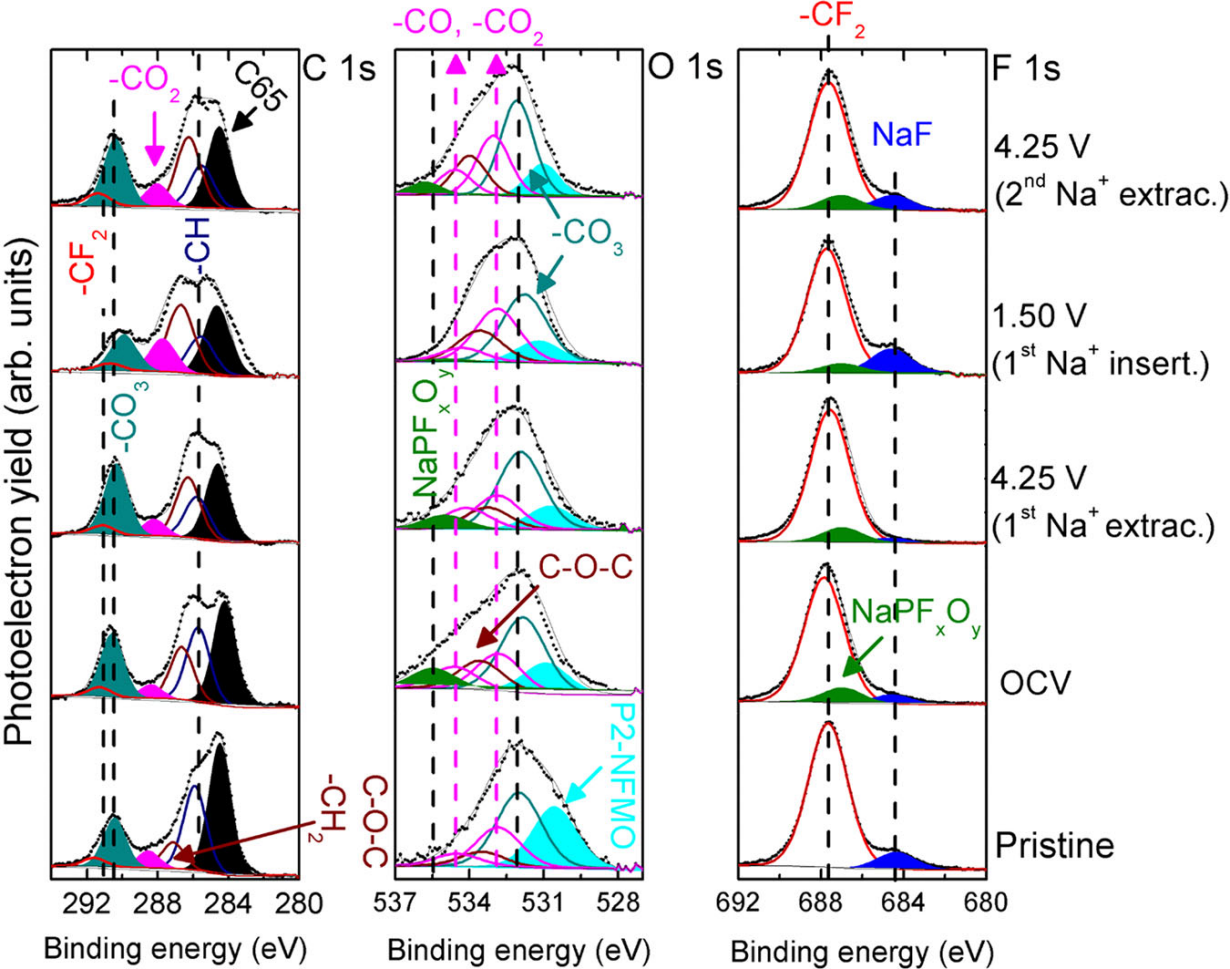
XPS spectra of P2-NFMO-LV electrodes. C 1s, O 1s, and F 1s photoemission lines of P2-NFMO-LV electrodes before cycling (pristine) and at different SOC (first Na^+^ extraction, first Na^+^ insertion, and second Na^+^ extraction) during electrochemical cycling as highlighted in Fig. S1.

The formation and stability of the outermost EEI of P2-Na_2/3_[Fe_1/2_Mn_1/2_]O_2_ electrodes cycled at different states of charge (SOC - as indicated in the galvanostatic profile of Supplementary Fig. S1) has been measured by means of XPS. Figure 4 shows the C 1s, O 1s, and F 1s photoemission lines while the binding energies of the observed species are collected in Supplementary Table S1.

First, the formation of the EEI is confirmed at OCV by the intensity decrease of electrode component photoelectron peaks: C65 component in the C 1s region (black line)^62^, P2-Na_2/3_[Fe_1/2_Mn_1/2_]O_2_ component in the O 1s region (cyan line)^44^ and -CF_2_ component from PVdF in the F 1s region (red line)^63^ (see also the concentration of the mentioned species in Supplementary Fig. S2). The EEI formation at OCV occurs by the reduction of solvents and salt decomposition, as also observed in other Na-based electrodes and attributed to the reductive nature of layered oxides and instability of metallic sodium^19,44–46,48^. Second, at further electrochemical cycling, the formed EEI is composed of C-O-C species, such as polyethylene oxide (PEO, (-CH_2_-CH_2_-O-)_*n*_)^64^ originated from direct polymerization of ethylene carbonate (EC) and diethyl carbonate (DEC)^65^, as well as NaCO_3_R (R = alkyl group/s) and Na_2_CO_3_ from EC and DEC reduction as shown in C 1s and O 1s spectra^45,66–68^. Indeed, the outermost surface region is mainly composed of Na_2_CO_3_ as confirmed by the Na 1s spectra (Supplementary Fig. S3). Besides the mentioned carbonaceous/oxygenated species, the F 1s spectrum reveals NaF formation due to the PVdF dehydrofluorination reaction^43^, as well as NaPF_6_ decomposition reactions^46,69^.

On the other hand, the first evidence of the EEI stability can be observed at a glance, note that the most significant difference is found between the XPS spectra of the pristine and OCV electrodes, while the XPS spectra from cycled electrodes are rather similar to the ones from OCV electrode, confirming that the surface composition of the electrodes does not undergo major changes upon electrochemical cycling (see also Supplementary Fig. S2, illustration of the concentration percentage of the electrode components). Indeed, the presence of stable components corresponding to the pristine electrode (C65 (C1s), P2-Na_2/3_[Fe_1/2_Mn_1/2_]O_2_ (O 1s), and -CF_2_ group of PVdF (F 1s)) is in agreement with the constant *R*_EEI_ values measured by EIS. The peak shape and intensity slightly vary during Na^+^ insertion, for example, the NaCO_3_R and NaF concentration increases, which is due to their partial dissolution in the organic carbonate-based electrode during Na^+^ extraction, and might be the responsible of the *R*_EEI_ drop observed below 2.1 V^43,67^. This has been previously reported for similar layered oxides (note that the Na_2−*x*_CO_3_R_*x*_ (*x* = 1) and NaF are highly soluble in organic carbonate-based electrolytes)^44,45^. It can be concluded that, although electrolyte oxidation/reduction is not expected in the P2-NFMO-LV electrode since the operating voltage window is inside the electrochemical stability window of the organic carbonate-based electrolyte^70^, a thin EEI is formed on the active material and conductive carbon already at OCV SOC, whose thickness and chemical composition slightly vary at different SOC. Indeed, the thin EEI cannot be detected by Fourier-transform Infrared spectroscopy (FTIR), as shown in Supplementary Fig. S4, where the FTIR spectra of the cycled electrodes are identical.

As a result, the overall increase of *R*_CT_ and *R*_elec_ in the second cycle with respect to the first cycle indicates that the Na^+^ insertion into the electroactive material is hindered and the P2-NFMO-LV electrode becomes a worse electronic conductor. Such variation in the electronic properties is nested in the structural changes, namely the increase of the *c* parameter, owing to the electrostatic repulsion between oxygen layers^15,20^; that ultimately results in an increase of the bulk electronic conductivity, as already observed for the Li-based LiCoO_2_ layered oxide^53,54^.

In addition, the intrinsic electronic structure of the involved phases has been assessed using DFT calculations, as detailed in the experimental section, to better understand the origin of this observation. The density of states (DOS) for P2-Na_2/3_[Fe_1/2_Mn_1/2_O_2_] and O2-Na_1/5_[Fe_1/2_Mn_1/2_]O_2_ model structures are shown in Supplementary Fig. S5a, b, respectively. The O2-type structure should be considered as a proxy of the more complex “*Z*”/OP4-type structure, which is the most plausible phase formed at high voltage as has been experimentally reported^16,20–23^. Using an actual OP4 structural model involves a very high computational cost because large supercells and a prohibitive number of combinatorial Na^+^/vacancy and Fe/Mn site distributions would need to be considered, which is beyond the scope of this work. In fact, a simple O2-based structural model is representative of the main possible orderings and local environments of the transition metals under the presence of Na^+^/vacancy octahedral sites within OP4-type structures. Such octahedral environments are the main local structural difference with respect to the prismatic environments found in the P2-phase. For the P2-type structure, the computed DOS reveals a valence band maximum populated with Mn states and the presence of a band gap of 0.5–0.8 eV between the valence and conduction bands (Supplementary Fig. S5a). In the case of the O2-type structure, the onset of the conduction band slightly increases by less than 0.3 eV with respect the P2 structure, and two discrete Fe spin-up states appear within the band gap yielding a pseudo-half-metallic character to the system (Supplementary Fig. S5b). Such differences between the electronic structures of P2- and O2-type model structures are not sufficiently large to suggest a noticeable qualitative difference in the intrinsic electronic conductivity between P2- and OP4-type phases. This is in stark contrast with the measured *R*_elec_ values that reveal the more insulating character of the Na^+^ extracted cathode electrode with respect to the more sodiated one. At this point, several factors should be considered upon the interpretation of the DFT results. Indeed, the calculated band gaps correspond to the active material while the EIS measurements correspond to the behavior of the whole electrode where the active material properties are deconvoluted. Therefore, the eventual changes in particle size, interface and/or exfoliation, staking faults, etc. appearing upon cycling are not considered by the DFT calculations; certainly, these factors influence the bulk electronic conductivity of the material.

In order to observe experimentally these possible changes that can affect the bulk electronic conductivity of the electrode, the measured *α* value of *C*_elec_ has been analyzed. *α* is the exponential factor of the electronic constant phase element (CPE) and for an ideal surface, *α* is equal to 1. Figure 3b summarizes the *α* values of the first two electrochemical cycles, and it shows an overall decrease of *α* during the electrochemical cycling (variations are observed in the phase transition regions). The *α* factor strongly depends on the surface homogeneity, roughness and degree of polycrystallinity^51^, so its continuous decrease is suggesting that the surface of the electrode is becoming more heterogeneous and/or the crystallinity is being loss upon electrochemical cycling. Since these aspects cannot be considered by the performed DFT calculations, and even if the P2- and O2-phases can be representative of the OP4-phase, this increase of the heterogeneity, roughness, and/or polycrystallinity of the active material might be the origin of the discrepancy between the DFT and EIS results.

### P2-Na_2/3_[Fe_1/2_Mn_1/2_]O_2_ cycled in the 2.0–4.0 V vs Na^+^/Na voltage window: P2-NFMO-SV electrode

One of the approaches to enhance the capacity retention of layered oxides is by controlling the operating voltage window, obtaining the best results in the 2.0–4.0 V vs Na^+^/Na window, as mentioned above^22,24,29,59,71^. Supplementary Fig. S6 shows that, after 45 cycles, the P2-NFMO-LV electrode delivers the same capacity as the P2-NFMO-SV electrode, although the initial capacity is 45 mAh g^−1^ higher. The capacity retention of both electrodes is illustrated in Supplementary Fig. S6b, and it can be clearly observed that the P2-NFMO-SV electrode exhibits higher capacity retention than the P2-NFMO-LV electrode. Therefore, in order to study the effect of the transport properties, and mainly the *R*_elec_, in the smallest operating voltage window, where the phase transition at high voltage is avoided (P2 solid-solution region, taken over the distorted P′2 phase at around 2.0 V), the same EIS methodology applied to the P2-NFMO-LV electrode has been carried out between 2.0 and 4.0 V (P2-NFMO-SV electrode). This approach helps to corroborate the effect of the high volume change of the “*Z*”/OP4-type structure in the transport properties and its role in the *R*_elec_ increase.

The Nyquist plot of the P2-NFMO-SV electrode at OCV (2.68 V vs Na^+^/Na, Supplementary Fig. S7) shows a very similar lineshape to the one from the P2-NFMO-LV electrode (Fig. 1). In fact, three semicircles at the same frequency regions are observed: HF region above 5 kHz (red region), MF region in the 5 kHz–10 Hz range (green region), and LF region below 10 Hz (blue region) with a sloping line at the lowest frequencies.

**Fig. 5:**
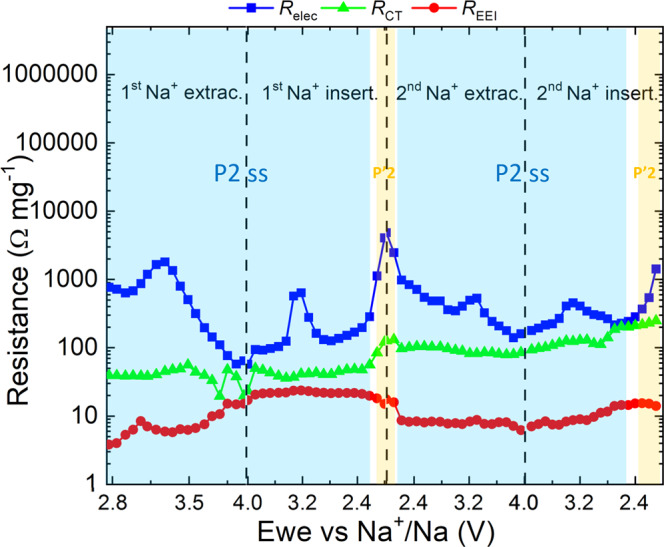
Fitted resistance values for the first two electrochemical cycles of the P2-NFMO-SV electrode. *R*_EEI_ (red points), *R*_CT_ (green triangle), and *R*_elec_ (blue square). The structural evolution highlighted as colored regions and labeled upon electrochemical cycling is included in the figure for guidance^15, 20^.

The impedance spectra of the P2-NFMO-SV electrode during first Na^+^ insertion and Na^+^ extraction at different SOC (see Nyquist plots of Supplementary Fig. S8) show that it follows the same behavior as the P2-NFMO-LV electrode. This means that the material becomes a better electronic conductor (*R*_elec_ decrease) during the Na^+^ extraction process, while, upon Na^+^ insertion, the active material exhibits the opposite trend, becoming a worse electronic conductor (see Fig. 5). Interestingly, *R*_elec_ is more stable than in the P2-NFMO-LV electrode during the first two cycles, which most probably is due to the blocking of the “*Z*”/OP4-type structure formation. However, at ~2.0 V vs Na^+^/Na an increment of the LF semicircle size (Supplementary Fig. S8c, pink curve) and the *R*_elec_ value (a yellow region in Fig. 5) is observed, owing to the fact that, at this voltage, the phase transition from ordered P2 to distorted P′2 occurs. The *R*_elec_ is reversible within this voltage range, indicating that the “*Z*”/OP4-type structure formation is the main responsible for the resistance increase/loos of the electronic conductivity.

*R*_CT_ (green triangles of Fig. 5) increment is also more stable in the P2-NFMO-SV electrode. The slight increase during the electrochemical cycling, likewise for the P2-NFMO-LV electrode, suggests that by avoiding the structural change at high voltage, the formation of grain boundaries, mosaic texture, exfoliation, and/or interfacial microstrains is reduced.

Finally, the *R*_EEI_ (red points of Fig. 5) trend is the same as for the P2-NFMO-LV electrode. The *R*_EEI_ drop observed at 2.1 V vs Na^+^/Na during the second Na^+^ extraction might be correlated with slight changes in the EEI surface, also observed in the second Na^+^ insertion on the P2-NFMO-LV electrode. Even so, after further cycling, the *R*_EEI_ stabilizes at a constant value.

Henceforth, avoiding the “*Z*”/OP4-type structure formation by reducing the operating voltage window, which is one of the strategies (note that by chemical tunning also the high voltage transition can be avoided)^30,31,33,34,39,40^, the electrode displays a high bulk electronic conductivity with stable *R*_elec_ values. The electronic conductivity loos due to the “*Z*”/OP4-type structure formation results due to the huge volume change that isolates the active particles from the conducting carbon and/or current collector, while triggering strong internal stress. In turn, the electronic conductivity is lost and/or reduced^19^, which ultimately influences the capacity fading.

### Further cycling of P2-NFMO-LV and P2-NFMO-SV electrodes

**Fig. 6:**
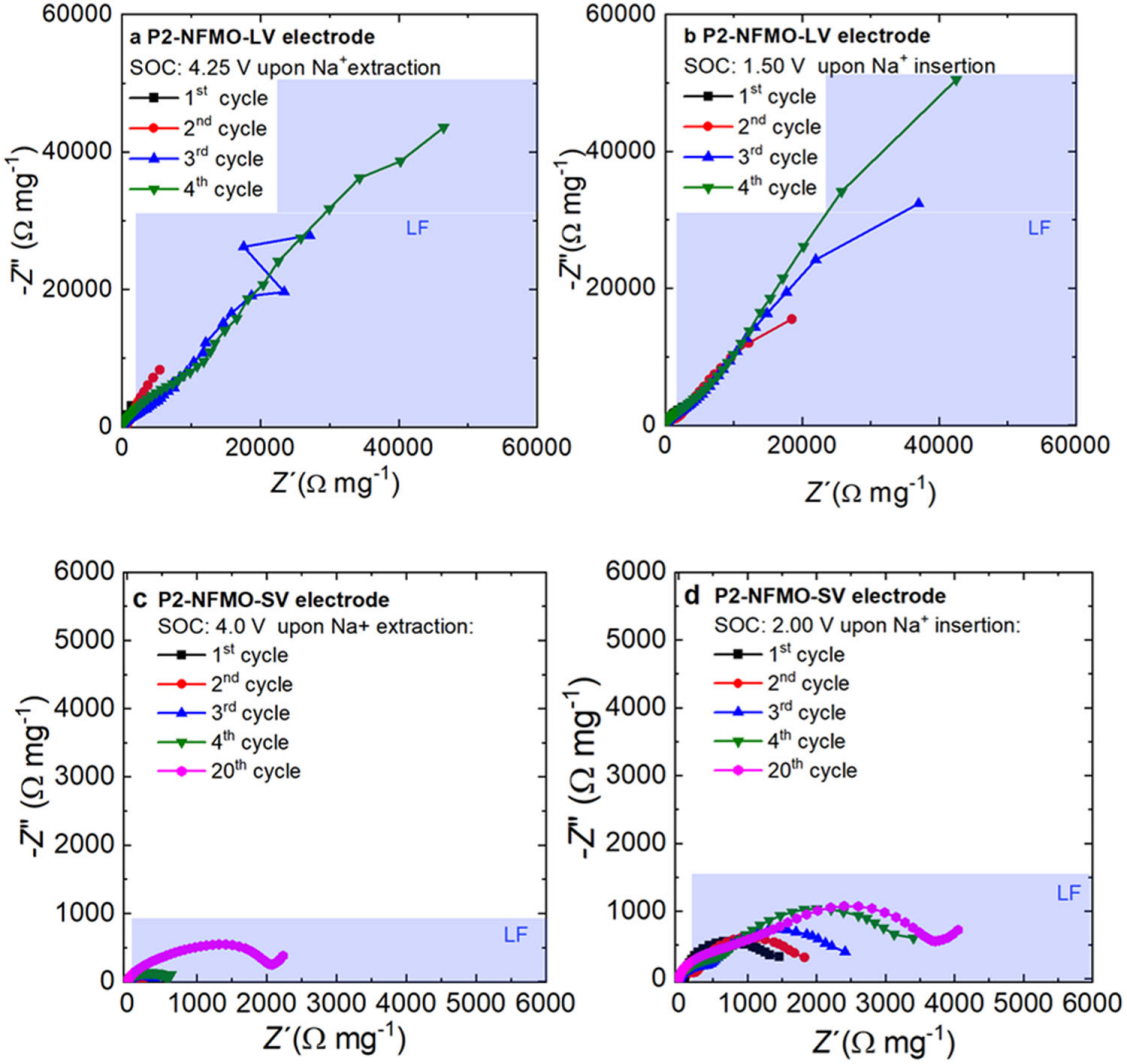
Nyquist plots of P2-NFMO-LV and P2-NFMO-SV electrodes upon electrochemical cycling. P2-NFMO-LV at **a** 4.25 V and **b** 1.50 V and P2-NFMO-SV electrode at **c** 4.0 V and **d** 2.0 V (vs Na^+^/Na) of the 1^st^ (black square), 2^nd^ (red point), 3^rd^ (blue triangle), 4^th^ (green inverted triangle), and 20^th^ cycle (magenta hexagon).

The impedance spectra have also been recorded after 1, 2, 3, 4, and 20 cycles for both electrodes (Fig. 6). Although the fits become more complicated after long cycling because of the large semicircle developed at LF (highlighted in blue in Fig. 6) which overlaps with the contribution from other processes at higher frequencies (*i.e.*, *R*_CT_ and *R*_EEI_), it can be observed that, in both electrodes (P2-NFMO-LV and P2-NFMO-SV), the overall resistance increases upon cycling. Nevertheless, the increment rate of the overall resistance in the P2-NFMO-SV electrode is much lower than for the P2-NFMO-LV electrode.

*R*_elec_ of P2-NFMO-LV shows a remarkable increase in the first four cycles. Meanwhile, the total resistance of the P2-NFMO-SV electrode is one order of magnitude smaller in the fourth cycle even if the phase transition at low voltage (P2 to P′2) is not avoided. Furthermore, in the twentieth cycle, the total resistance of the P2-NFMO-SV electrode is still lower than that of the P2-NFMO-LV electrode in the fourth cycle.

Therefore, although it is considered that the structural evolution of P2-Na_2/3_[Fe_1/2_Mn_1/2_]O_2_ is reversible, the XRD data acquired after more than 75 cycles of the P2-NFMO-LV electrode clearly show that the full-width at half-maximum (FWHM) is drastically increasing upon electrochemical cycling (Supplementary Fig. S9). The P2-Na_2/3_[Fe_1/2_Mn_1/2_]O_2_ (002) reflection of the cycled electrode until 4.25 V (>75 cycles) exhibits a much larger FWHM value than the pristine one (0.23° vs 0.09°). These phase transitions induce a [TMO_2_]-gliding, which for the P2- to OP4-phase transition, the interlayer distance is reduced from 5.65 to 5.30–5.05 Å, while in the structural change at 2.0 V (P2 to P′2) the difference is 0.10 Å lower for P′2-type^24^. As it was mentioned, these phase transitions lead to an unavoidable volume change and a significant increase of the interfacial microstrain, staking faults formation, and/or exfoliation of the layers as confirmed by previous reports on the grounds of SEM/TEM experiments^25,26,61^. These structural modifications are the responsibility of the decrease of electronic conductivity and greatly contribute to the reported capacity loss. On the other hand, although in the first cycles the phase transition at low voltage (from P2 to P′2) is reversible, it is clearly observed that upon further cycle (see Fig. 6c, d), the overall resistance of the electrode also increases (lower than in P2-NFMO-LV) that at long-term cycling will be an issue, also contributing in the capacity fading. Hence, avoiding the phase evolution, for example by using a reduced operating voltage window (2.0–4.0 V vs Na^+^/Na), better capacity retention is obtained, although lower initial capacity values are delivered (Supplementary Fig. S6), due to the transport properties maintain stable.

## Conclusions

EIS experiments show that the responsible, in terms of transport properties, of the poor capacity retention of P2-Na_2/3_[Fe_1/2_Mn_1/2_]O_2_ layered oxide cathode material is the formation of the “Z”/OP4 phase that induces the loss of the bulk electronic conductivity of the electrode. DFT calculations rule out the possibility that such observation is due to significant changes in the intrinsic electronic structure of the bulk materials during cycling. Since the XPS studies show that the EEI is rather stable and the DFT results suggest that no significant changes should be expected for different orderings of this structure, such bulk electron conductivity loss is most probably due to the large volume changes occurring in the active material when cycling above 4.0 V vs Na^+^/Na where a P2- to “*Z*”/OP4-type phase transition occurs. These volume changes lead to interfacial microstrain, staking faults formation, and/or exfoliation of the layers that, besides reducing the bulk electron conductivity, lead to the progressive isolation of the active particle with respect to the conducting carbon and current collector. When the high cut-off voltage is set to avoid the structural change, the electronic resistance is maintained since the [TMO_2_]-layers are not glided, hence, the P2-Na_2/3_[Fe_1/2_Mn_1/2_]O_2_ electrode exhibits much better structural reversibility, as well as reversible resistance values and more stable transport properties and in turn better capacity retention. Such results might be extrapolated to other P2-layered oxides which, despite exhibiting good theoretical capacity when cycled up to 4.3 V vs Na^+^/Na, they should not be used as a high voltage cathode electrodes in order to keep good capacity retention and excellent cycle life for the SIB.

## Methods

### Synthesis of the active cathode material

P2-Na_2/3_[Fe_1/2_Mn_1/2_]O_2_ was synthesized by the solid-state method. First, stoichiometric amounts of Na_2_CO_3_·H_2_O (99.5%, Sigma Aldrich), Fe_2_O_3_ (99%, Alfa Aesar), and Mn_2_O_3_ (98%, Alfa Aesar) were mixed in a mortar for 2 h. The mixed powder was compressed in pellets and heated up to 900 °C for 12 h under air atmosphere followed by liquid nitrogen quenching^16^. The obtained sample was transferred and stored in an argon-filled glove box (MBraun, H_2_O and O_2_ < 1 ppm) in order to avoid any contact with the atmosphere.

### Structural and morphological characterization

The structural characterization of the synthesized active cathode material was performed by powder XRD using a Bruker Advance D8 instrument with copper radiation (Cu K*α*_1,2_ λ = 1.5406 Å, 1.5444 Å). The powder XRD pattern was refined by the Le Bail method and shows a pure P2-type structure with …AABBAA… staking sequence (Supplementary Fig. S10). The morphology was analyzed using SEM (Quanta 200 FEG-FEI model) operated at 30 kV. The SEM images show that most particles crystalize as hexagonal platelets of 5–10 µm of diameter (Supplementary Fig. S11).

### Electrochemical characterization

Electrodes were prepared by mixing 80% of P2-Na_2/3_[Fe_1/2_Mn_1/2_]O_2_ active material, 10% carbon Super C65 (Timcal C-Nergy-TM), and 10% PVdF (Solef® Arkema Group) dissolved in N-methyl-2-pyrrolidone (NMP—Sigma Aldrich). The slurry was cast on battery-grade aluminum foil and dried under vacuum overnight at 120 °C. 11 mm electrodes were pressed at 5 tons for 1 min before assembling cells in an argon-filled glove box (MBraun, H_2_O and O_2_ < 1 ppm). The galvanostatic experiments were carried out in CR2032 type coin-cells, using P2-Na_2/3_[Fe_1/2_Mn_1/2_]O_2_ electrodes as a working electrode and metallic sodium disk (99.8% Acros Organics) as a counter electrode, glass fiber (Whatman GF/D) as separator and 1 M NaPF_6_ in EC:DEC in a 1:1 wt% (Acros Organics) as electrolyte. The experiments were carried out in a Maccor Series 4000 battery tester in two operating voltage windows: (*i*) 1.5–4.25 V (P2-NFMO-LV) and (*ii*) 2.0–4.0 V (P2-NFMO-SV) vs Na^+^/Na, at 0.05C C-rate (1C = 260 mA g^−1^, which theoretically corresponds to the exchange of one Na^+^). EIS experiments were performed in three electrode Swagelok-T-type cells. Metallic sodium disks were employed as counter and reference electrodes, and the same electrolyte as for the galvanostatic experiments was used (1 M NaPF_6_ in EC:DEC in a 1:1 wt%). The experiments were carried out at room temperature in the same two operating voltage windows used for the galvanostatic tests: (*i*) 1.5–4.25 V (P2-NFMO-LV) and (*ii*) 2.0–4.0 V (P2-NFMO-SV) vs Na^+^/Na in a VMP3 potentiostat (Bio-Logic) through potentiostatic intermittent titration technique (PITT) where the potential of the electrode was controlled with a 45 mV step scan. A sinusoidal perturbation of 5 mV was applied in the frequency range of 100 kHz–5 mHz and before EIS data were taken a 4 h of equilibrium condition was set at a constant potential. Impedance dispersion data (133 for MFMO-LV and 76 for NFMO-SV impedance spectra in total) were fitted by Boukamp’s Equivalent Circuit software^72^. This model is based on the following parameters: (*i*) Na^+^ resistance across the electrolyte (*R*_sol_), (*ii*) resistance and capacitance of the EEI (*R*_EEI_ and *C*_EEI_), (*iii*) *R*_CT_ and *C*_DL_, (*iv*) *R*_elec_ and *C*_elec_, (*v*) Warburg diffusion (*Z*_w_) related to the solid-state diffusion of Na^+^, and finally (*vi*) intercalation capacity (*C*_i_) due to the charge accumulation (see top of Supplementary Fig. S12)^51,55,56^. The resistance and capacitance of each process are connected in parallel. Additionally, in order to take into account any deviation from an ideal material such as surface inhomogeneity, roughness or degree of polycrystallinity, the *C* elements and *Z*_w_ have been replaced by CPE. In order to illustrate the goodness of the fits, some fitted EIS spectra are shown as examples in Supplementary Fig. S12a (P2-NFMO-LV electrode) and S12b (P2-NFMO-SV electrode), while the obtained values from the fit are collected in Supplementary Tables S2 and S3, respectively.

### Characterization of EEI

The EEI was studied, by means of XPS using a Phoibos 150 spectrometer (Specs GmbH), at several SOC (cycled at 0.05C): pristine, OCV, 1^st^ Na^+^ extraction and insertion and 2^nd^ Na^+^ extraction states as highlighted in the galvanostatic profile of Supplementary Fig. S1. The EEI composition and stability are followed by analyzing the C 1s, O 1s, F 1s, and Na 1s photoemission lines. Since the Auger peaks and photoelectron peaks were overlapped in some cases, two different X-ray sources were used: a non-monochromatic Mg K_α_ source (*hν* = 1253.6 eV), which was applied for C 1s and O 1s photoelectron peaks and a non-monochromatic Al K_α_ source (*hν* = 1486.6 eV) for all photoemission lines (C 1s, F 1s, and Na 1s). The electrodes were stopped at the selected SOC, rinsed with DEC, and dried before being inserted into the XPS vacuum chamber by means of an argon-filled transfer system, never exposing the electrodes to air. High-resolution scans at low potential were acquired at 100 W, 20 eV pass energy, and 0.1 eV energy step. Charging effects were compensated by flood gun at 10 μA and 1.5 V in order to correct slight variations of the binding energy^73^. Calibration of the binding energy was performed using the C 1s graphitic signal as a reference at 284.4 eV. The recorded spectra were fitted by CasaXPS software using a nonlinear Shirley-type background and a Voigt profile (70% Gaussian and 30% Lorentzian)^74^. The composition of the EEI and subsurface region has also been evaluated by means of FTIR using an Agilent Technologies Cary 630 benchtop system placed inside an inert atmosphere glove box. The electrodes were rinsed in DEC prior to FTIR experiments to remove electrolyte salt traces.

### Theoretical methods

DFT calculations have been performed within the Vienna ab initio simulation package^75,76^, employing the PBE functional. In order to describe the localized nature of Fe and Mn 3d states, the DFT + U scheme of Dudarev *et al*. ^77^ has been applied, with *U* = 4.0 eV for Fe and Mn atoms as suggested in the literature^78,79^. All calculations were spin-polarized, starting from a high-spin configuration. PBE-based projector augmented wave potentials^80^ were used to replace core electrons, whereas we treated explicitly the Na (3s), Fe (3p, 3d, 4s), Mn (3p, 3d, 4s), and O (2s, 2p) electrons as valence electrons and their wave-functions were expanded in plane-waves with cut-off energy of 600 eV. The irreducible Brillouin zone was sampled using a Monkhorst–Pack grid^81^ with 7 × 7 × 1 k-point sampling per (1 × 1) unit cell. Ground state energies for every configuration were computed allowing the lattice parameters, cell shape, and atomic positions to relax with a residual force threshold of 0.02 eV Å^−1^. It is known that in binary or ternary transition metal P2-compounds, the presence of Mn atoms can suppress long-range Na^+^/vacancy orderings^42,69,82^. For the P2-structure, the Na^+^/vacancy ordering used was previously found for a range of other P2-phases with 2/3 Na^+^ content: P2-Na_2/3_CoO_2_^83^, P2-Na_2/3_Fe_2/3_Mn_1/3_O_2_^84^, and P2-Na_2/3_Mn_2/3_Ni_1/3_O_2_^78^. This ordering consists of a zig-zag pattern of Na1 and Na2 sites arranged in triangular units, involving a supercell with in-plane lattice vectors *a*_super_ = 3*a* + 2*b* and *b*_super_ = 2*a* + 3*b*, containing 24 formula units with 16 Na, 12 Fe (III), 12 Mn (III) and 8 Mn (IV) and 48 O atoms. On the other hand, three different Na^+^/vacancy orderings were considered for the O2-structure, one with Na atoms in rows, the other in a hexagonal ordering, and the last one mixing both of them. In this case, we have used a supercell containing 36 formula units with 8 Na, 8 Fe (III), 10 Fe (IV), 18 Mn (IV), and 72 O atoms. All of them are in a high spin configuration, with a total magnetic moment of 100.0 and 134.0 for the P2- and O2- structures, respectively. The Na^+^/vacancy ordering assumed in this work should be considered as a low-energy, reasonable approximation, since from a computational viewpoint, the modeling of such arrangements would require large supercells which are unaffordable at the DFT level. For the Fe/Mn ordering in both oxides layers, we have thoroughly sampled a range of local structures within the considered supercell. In particular, for each investigated Na^+^/vacancy orderings, 50 different configurations were randomly generated and computed their DFT energy, in order to choose the most stable ones. The magnetic moment was used to distinguish the oxidation state of Mn and Fe. Finally, the DOS was calculated, to obtain the bandgap of the structures. In order to accurately determine Fermi energies, single-point DOS calculations were performed with twice the k-point density 14 × 14 × 2 per (1 × 1) unit cell as compared to geometrical optimization calculations.

## Supplementary information

Supplementary Information

Supplementary Data 1

Supplementary Data 2

Description of Additional Supplementary Files

## Data Availability

The optimized atomic coordinates of the P2- and O2-phases are included in the Supplementary Data 1 and 2 files, respectively. Restrictions apply to the availability of the datasets generated during and/or analyzed during the current study, and so are not publicly available. Data are however available from the authors upon reasonable request and with permission of CIC energiGUNE and HIU Batteries.

## Code availability

Computer codes used during the current study are available from Dr. O. Lakuntza or Dr. J. Carrasco upon reasonable request and with permission of CIC energiGUNE.
