# Supplementary material for: Role of the voltage window on the capacity retention of P2-Na2/3[Fe1/2Mn1/2]O2 cathode material for rechargeable sodium-ion batteries
Source: Commun Chem. 2022 Feb 1;5:11. doi: 10.1038/s42004-022-00628-0 (PMC9814619; doi:10.1038/s42004-022-00628-0)
Supplement: Supplementary file 1 — Supplementary Information [file 42004_2022_628_MOESM1_ESM.pdf]

# Supporting Information

## Role of the voltage window on the capacity retention of P2-$Na_{2/3}[Fe_{1/2}Mn_{1/2}]O_2$ cathode material for rechargeable sodium-ion batteries

Maider Zarrabeitia†,[a] Francesco Nobili,[b] Oier Lakuntza,[a] Javier Carrasco,[a] Teófilo Rojo,[a,c] Montse, Casas-Cabanas,[a,d] Miguel Ángel Muñoz-Márquez*,‡ [a]

[a] Dr. M. Zarrabeitia, Dr. O. Lakuntza, Dr. J. Carrasco, Prof. T. Rojo, Dr. M. Casas-Cabanas, Dr. M.A. Muñoz-Márquez
Centre for Cooperative Research on Alternative Energies (CIC energiGUNE), Basque Research and Technology Alliance (BRTA)
Alava Technology Park
Albert Einstein 48, 01510 Vitoria-Gasteiz, Spain
E-mail: miguel.munoz@unicam.it

[b] Dr. F. Nobili
School of Science and Technology – Chemistry Division
University of Camerino
Via Madonna delle Carceri, 62032 Camerino, Italy

[c] Prof. T. Rojo
Departamento de Química Inorgánica
Universidad del País Vasco UPV/EHU
P.O. Box 664, 48080, Leioa, Spain

[d] Dr. M. Casas-Cabanas
IKERBASQUE, Basque Foundation for Science
María Díaz de Haro 3, 48013, Bilbao, Spain

[†] current address: Helmholtz Institute Ulm (HIU), Helmholtzstrasse 11, 89081 Ulm & Karlsruhe Institute of Technology (KIT), P.O. Box 3640, 76021 Karlsruhe, Germany
[‡] current address: School of Science and Technology – Chemistry Division, University of Camerino, Via Madonna delle Carceri, 62032 Camerino, Italy

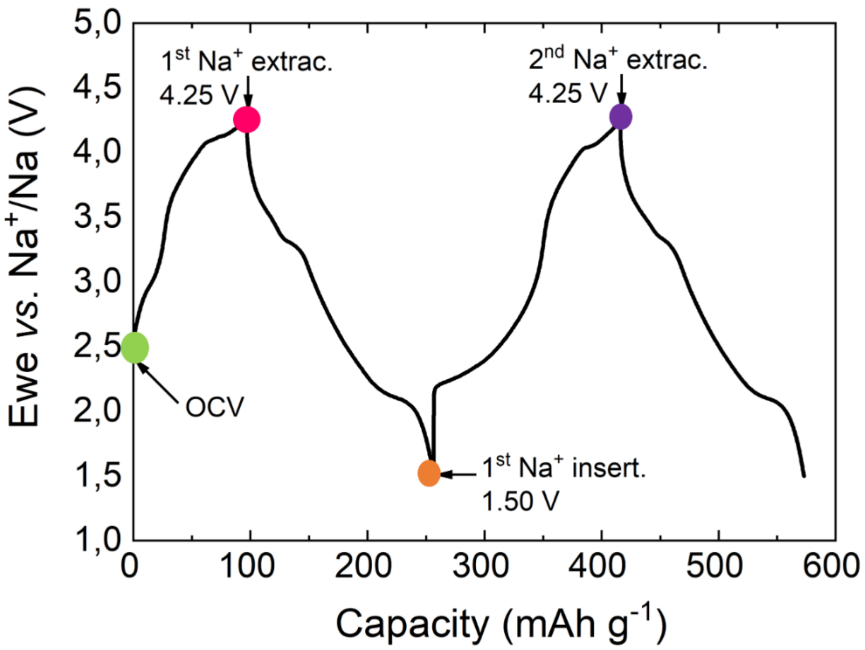


**Fig. S1:** Galvanostatic profile of P2-NFMO-LV electrode during the first cycle in the voltage window of 1.5 – 4.25 V vs $Na^+$/Na at 0.05C. The XPS measurements are performed in the pristine electrode and in the states of charge highlighted by color points in the galvanostatic curve.

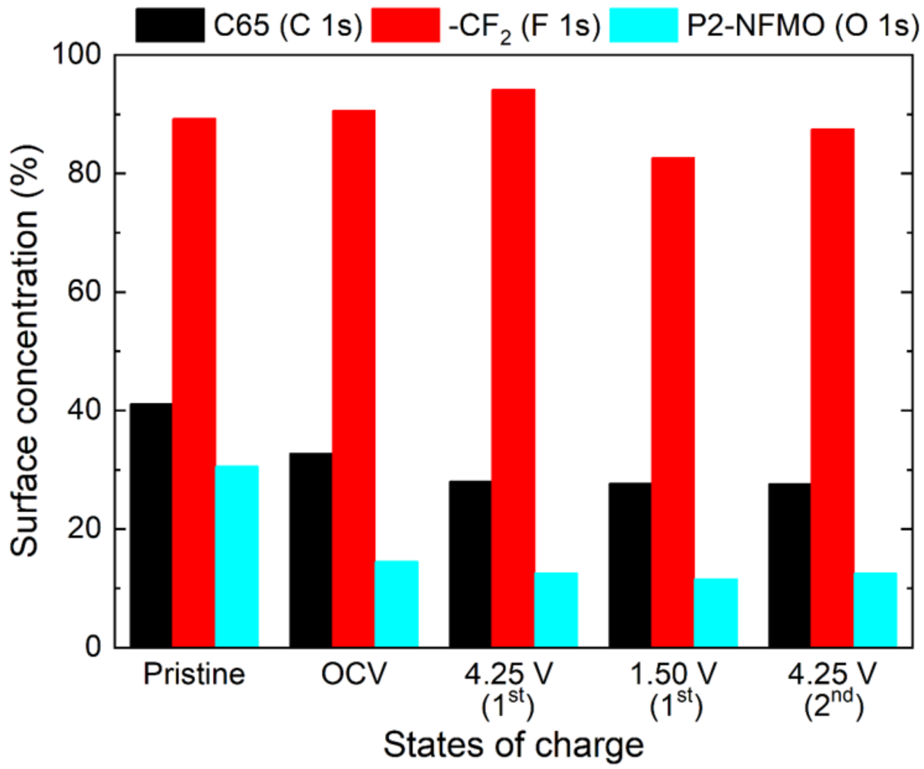


**Fig. S2:** Concentration percentage of the electrode component, *i.e.* conductive additive – C65 (black), binder – PVdF (red), active material -P2-NFMO (cyan) at different SOC.

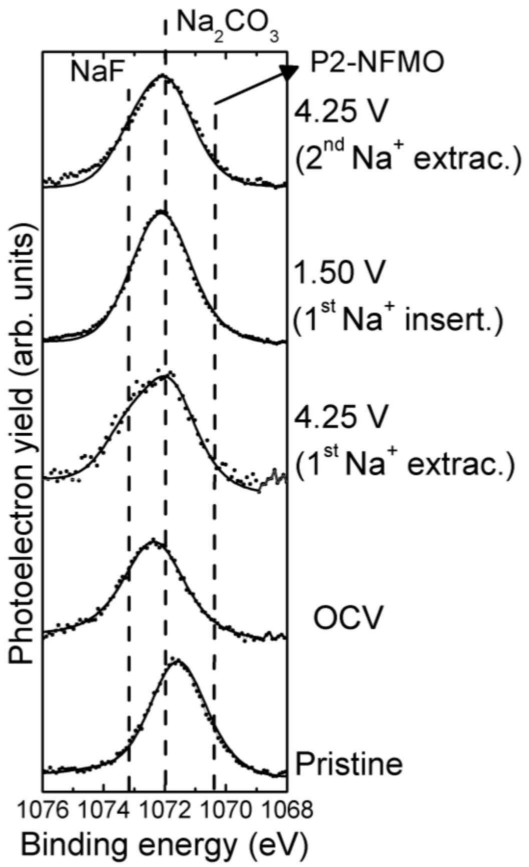


**Fig. S3:** Na 1s photoemission line of P2-NFMO-LV electrodes before cycling (pristine) and at different SOC during electrochemical cycling as highlighted in Fig. S1. It should consider that Na 1s photoelectron provides information about the outermost surface region since the detected photoelectrons have low kinetic energy and therefore a short inelastic mean free path.

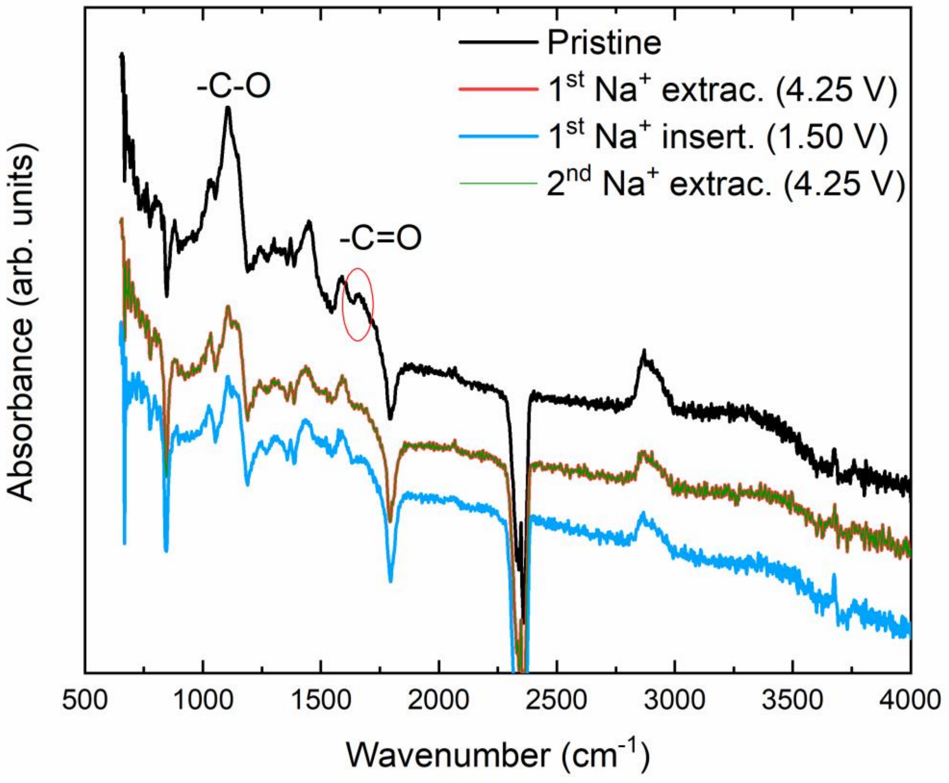


**Fig. S4:** FTIR spectra of pristine (black line) and cycled electrodes at SOC of 1st $Na^+$ extraction (red line), 1st $Na^+$ insertion (blue line) and 2nd $Na^+$ extraction (green line).

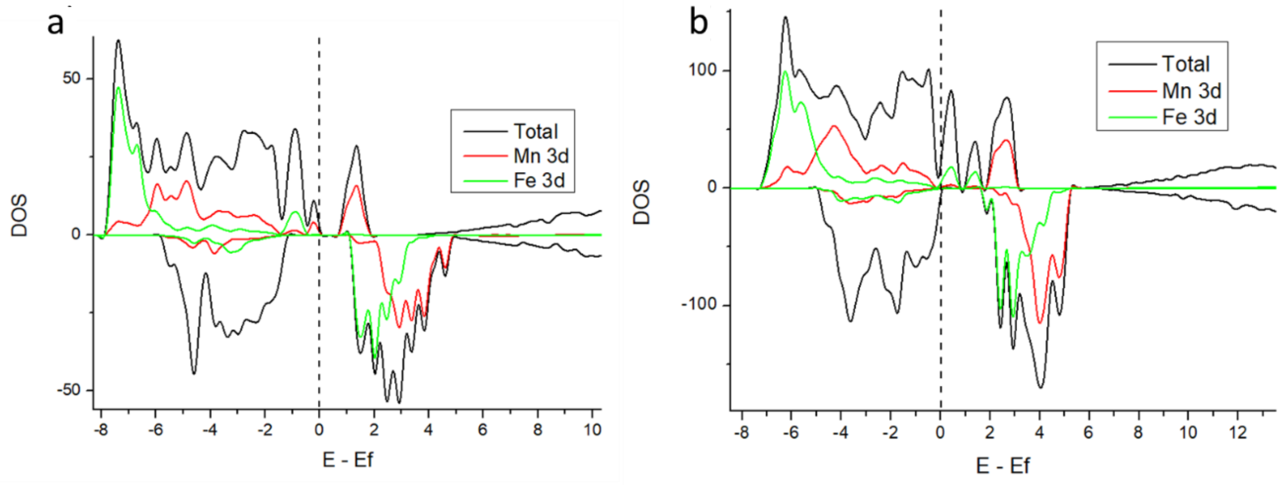


**Fig. S5:** DOS of **a** P2-$Na_{2/3}[Fe_{1/2}Mn_{1/2}]O_2$ and **b** O2-$Na_{1/5}[Fe_{1/2}Mn_{1/2}]O_2$. The Fermi level is set to zero.

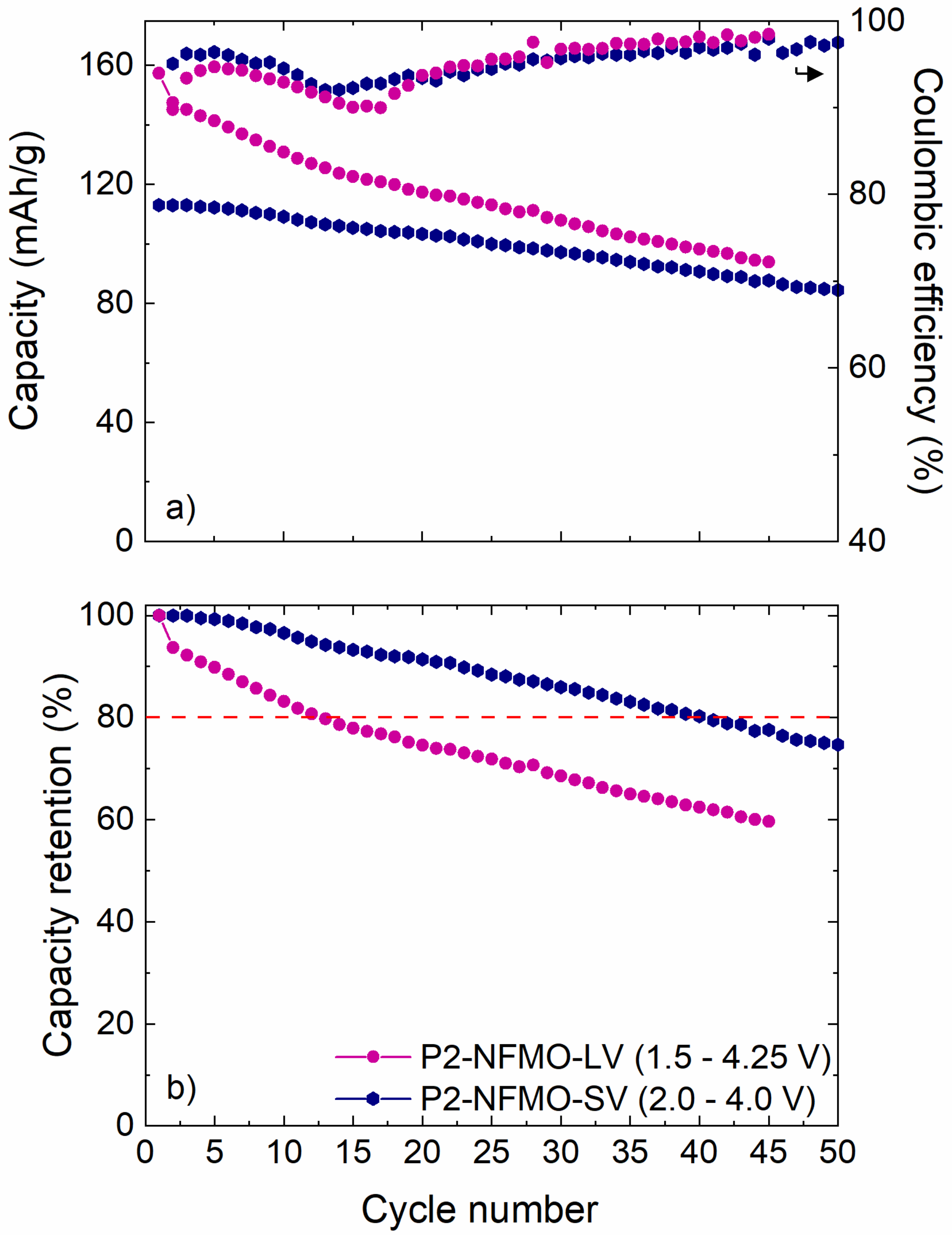


**Fig. S6: a** $Na^+$ inserted capacity and Coulombic efficiency vs cycle number and **b** capacity retention (considering 1st insertion capacity as reference) of P2-NFMO-LV (purple point) and P2-NFMO-SV (blue hexagon) electrodes. Note that the 80% of the capacity is loss after 12 cycles in the P2-NFMO-LV electrodes (highlighted by red dash line).

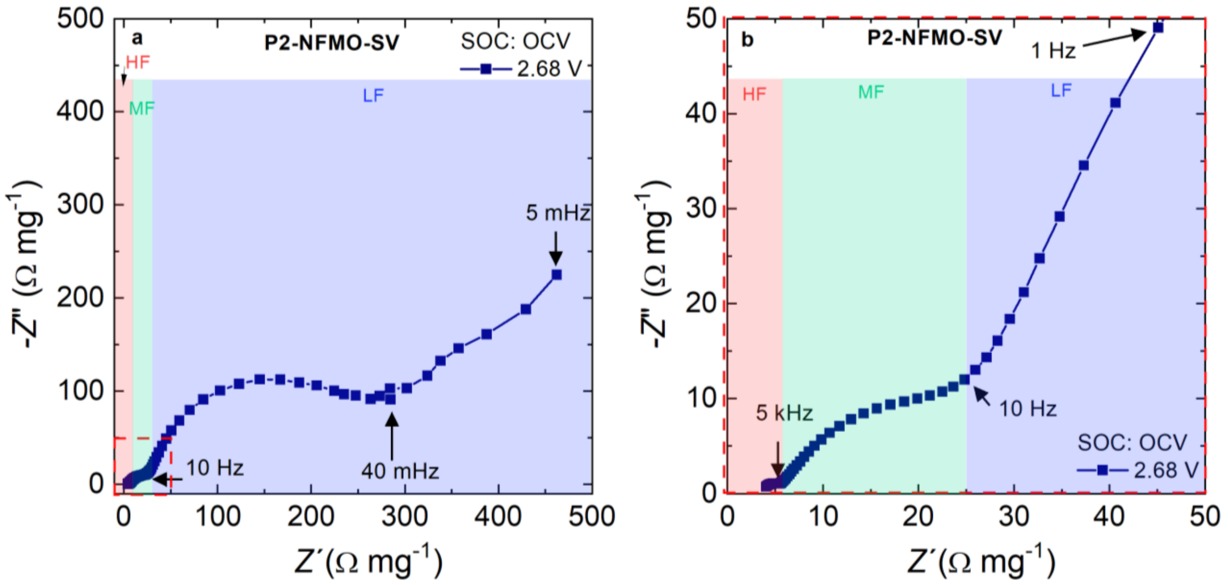


**Fig. S7:** Nyquist plot of P2-NFMO-SV electrode at OCV (2.68 V vs $Na^+$/Na). The impedance data from **a** 100kHz to 5mHz and **b** 100 kHz to 1 Hz. The frequency regions are highlighted (HF-red, MF-green and LF-blue).

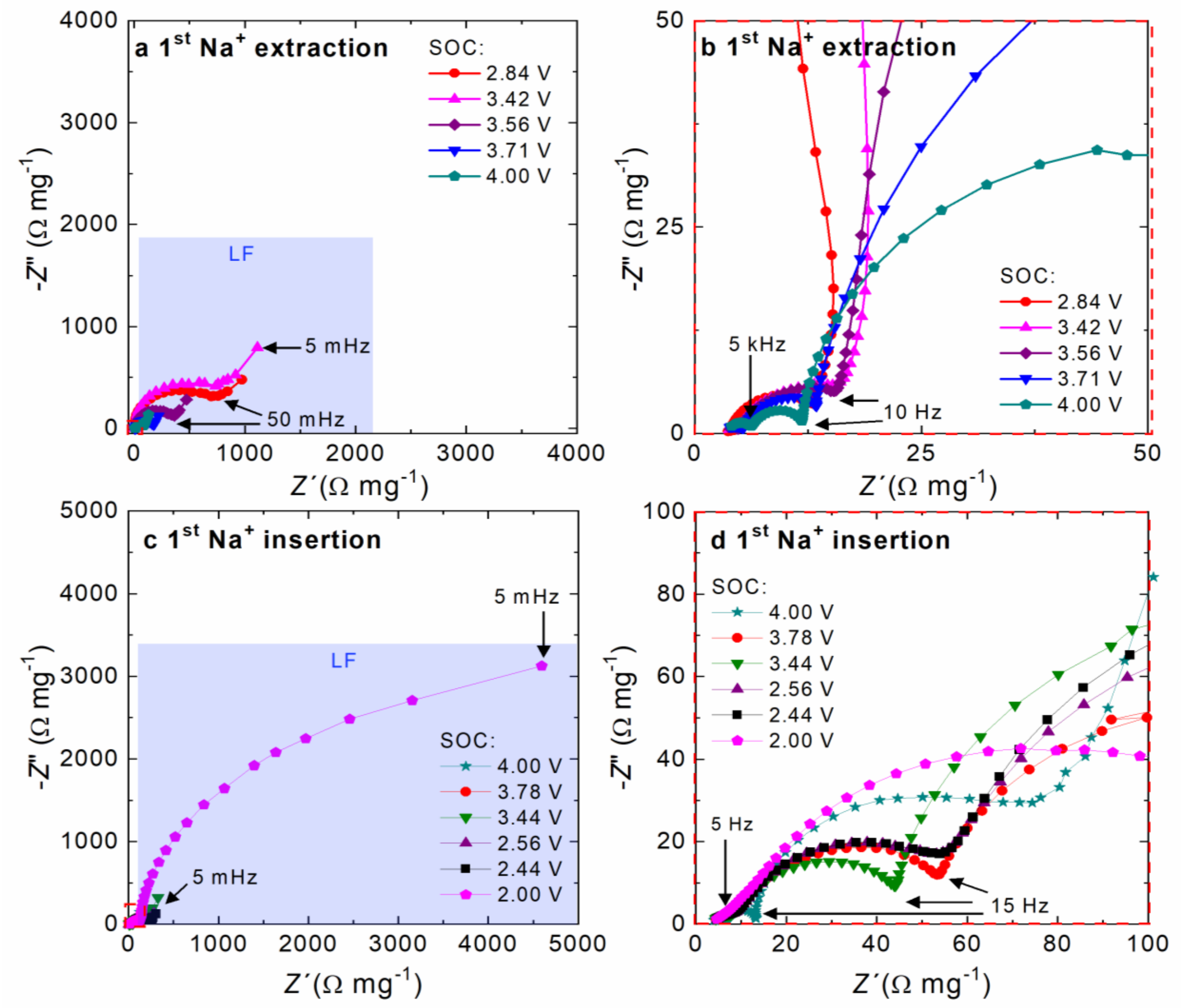


**Fig. S8:** Nyquist plots of P2-NFMO-SV electrode during first $Na^+$ extraction: at 2.84 V (red point), 3.42 V (magenta triangle), 3.56 V (purple rhombus), 3.71 V (blue inverted triangle) and 4.00 V (green pentagon) in the frequency range from **a** 100 kHz to 5 mHz and **b** 100 kHz to 1 Hz; and first $Na^+$ insertion: at 4.00 V (dark cyan star), 3.78 V (red point), 3.44 V (green inverted triangle), 2.56 V (purple triangle), 2.44 V (black square) and 2.00 V (magenta pentagon) in the frequency range from **c** 100 kHz to 5 mHz and **d** 100 kHz to 1 Hz.

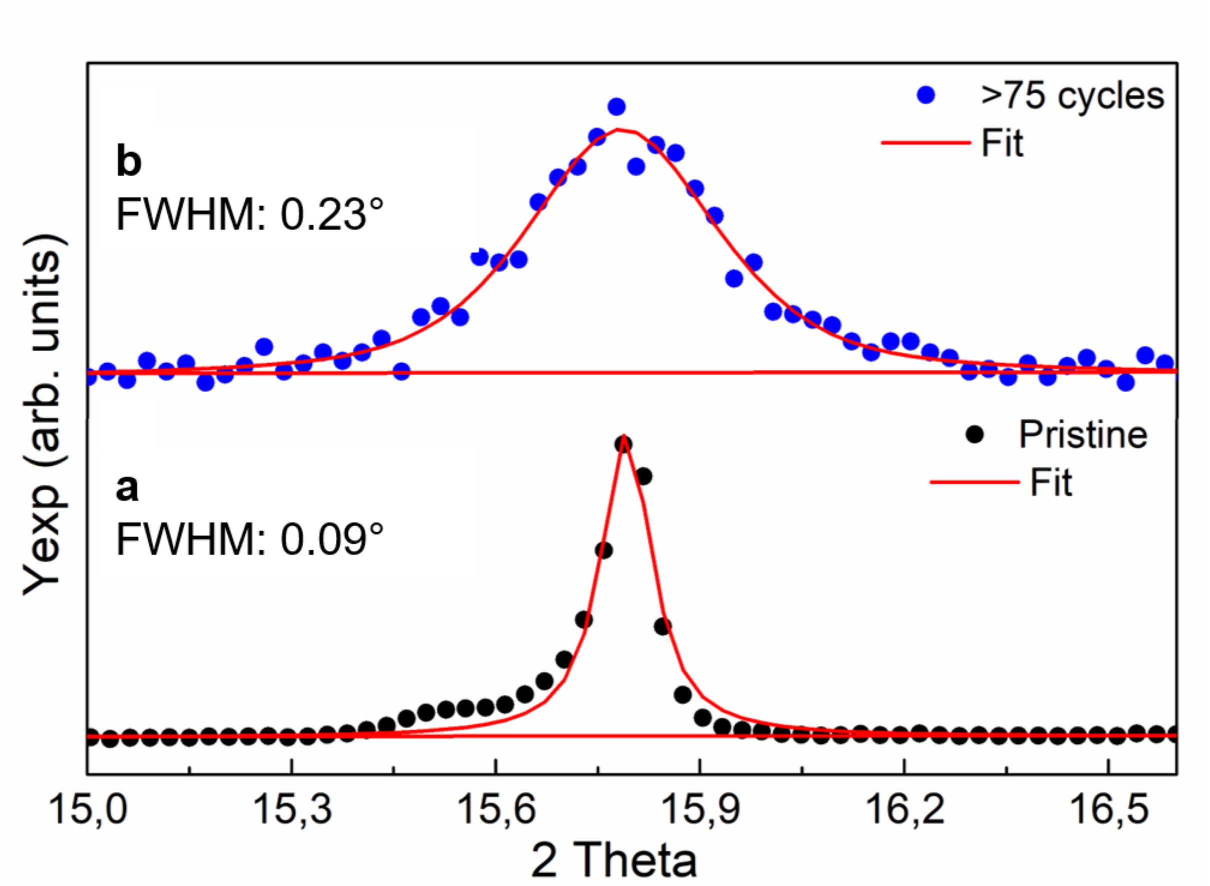


**Fig. S9:** Fit and the FHWM value of (002) reflection of P2-$Na_{2/3}[Fe_{1/2}Mn_{1/2}]O_2$ refined by FullProf software. **a** Pristine and **b** after >75 cycles in the operating voltage range of 1.5 - 4.25 V vs $Na^+$/Na.

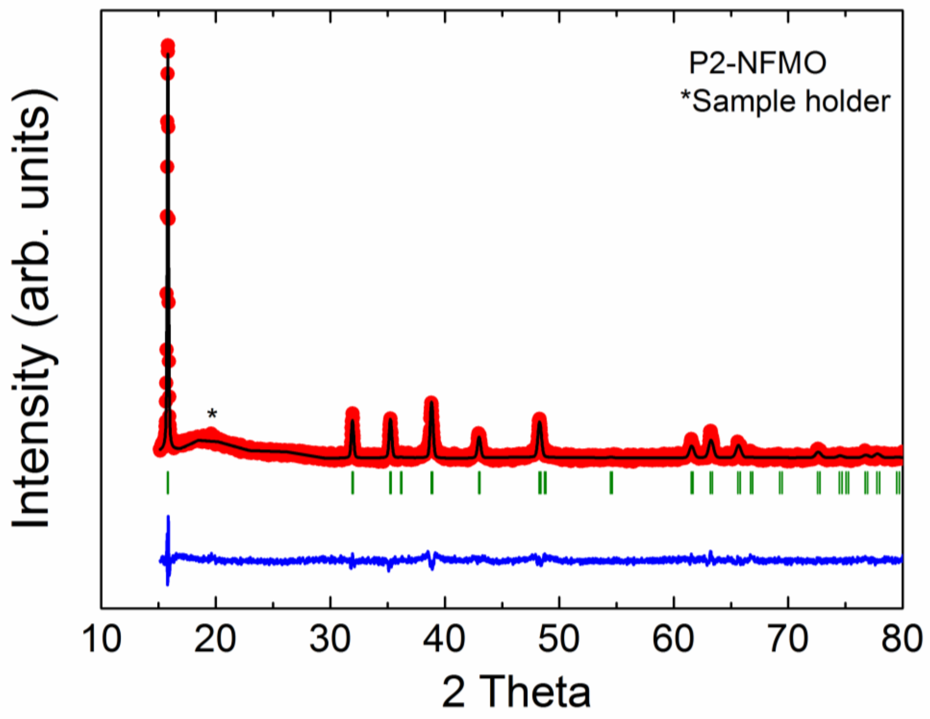


**Fig. S10:** Powder XRD of P2-$Na_{2/3}[Fe_{1/2}Mn_{1/2}]O_2$ refined by Le Bail method. Experimental (red point), calculated (black line), difference (blue line) and Bragg position (green vertical bar). * Diffraction lines from sample holder.

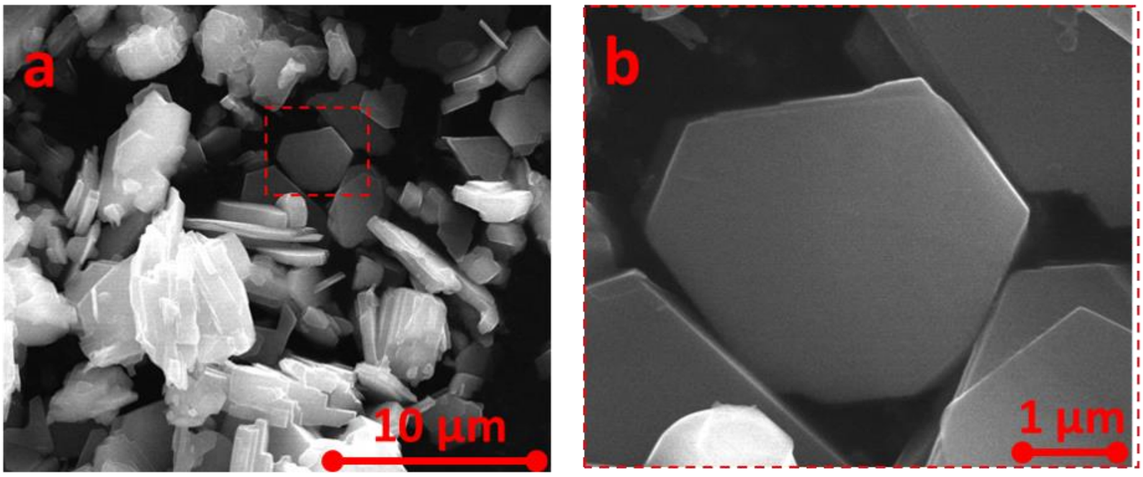


**Fig. S11:** SEM images of P2-$Na_{2/3}[Fe_{1/2}Mn_{1/2}]O_2$ powder acquired with **a** 6000 and **b** 30000 times magnification.

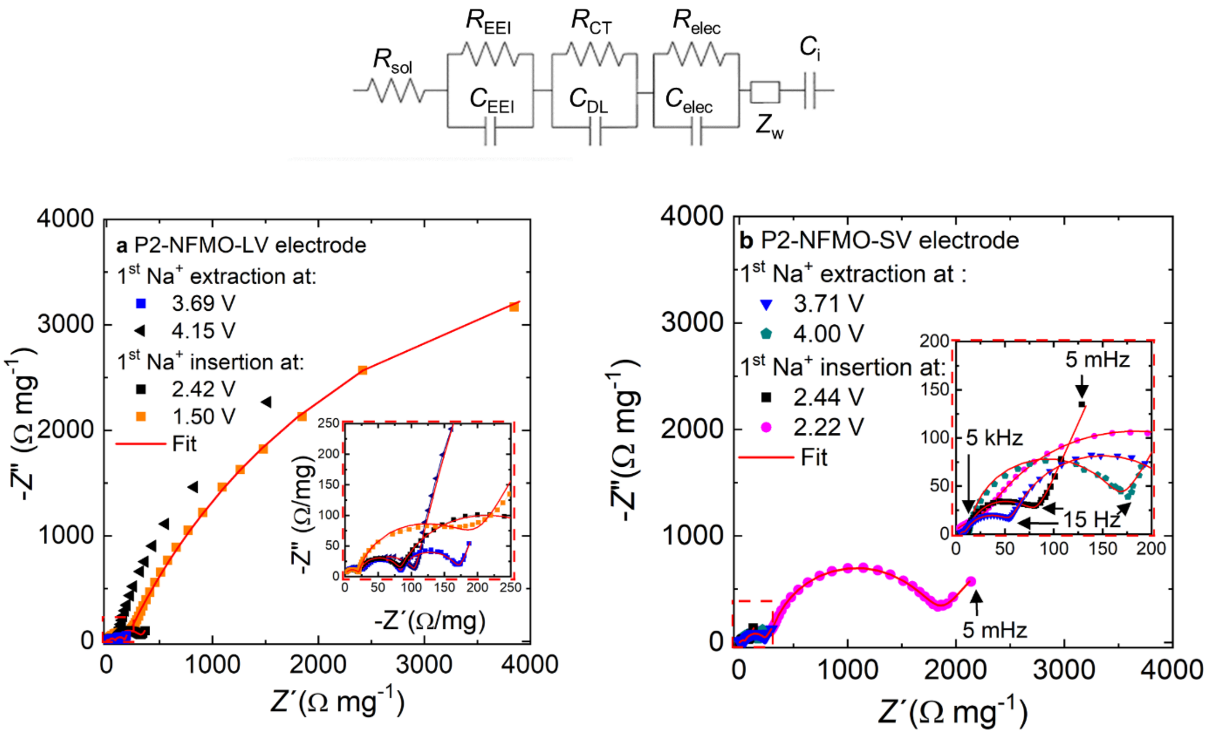


**Fig. S12:** The fits of the impedance data of **a** P2-NFMO-LV electrode (at 3.69 V – blue square and 4.15 V – black triangle upon $Na^+$ extraction and at 2.42 V – black square and 1.50 V – orange square upon $Na^+$ insertion *vs*. $Na^+$/Na, respectively) and of **b** P2-NFMO-SV electrode (at 3.71 V – blue inverted triangle and 4.00 V- dark cyan pentagon upon $Na^+$ extraction and at 2.44 V – black square and 2.00 V – magenta point upon $Na^+$ insertion vs $Na^+$/Na, respectively). The inset shows a zoom 100 kHz to 1 Hz and the used equivalent circuit on the top.

**Table S1:** Binding energy of the species that form the EEI.

| *Species* | *C 1s* | *O 1s* | *F 1s* | *Na 1s* |
|---|---|---|---|---|
| **C65** | 284.4 eV | | | |
| **P2-$Na_{2/3}[Fe_{1/2}Mn_{1/2}]O_2$** | | ~530.5 eV | | |
| **C-O-C (*i.e.* PEO)** | 286.3 eV | 533 eV | | |
| **$NaCO_3R$ (R=alkyl group)** | 287 – 288 eV | 532.5 & 534 eV | | |
| **$Na_2CO_3$** | 290 – 291 eV | ~532 eV | | 1072 eV |
| **$NaPF_xO_y$** | | 534 eV | 687 eV | |
| **NaF** | | | ~685 eV | |
| **PVdF** | 291 eV | | ~688 eV | |

**Table S2:** Values from the fits by Boukamp´s software of the impedance data at 3.69 V, 4.15 V, 2.42 V and 1.50 V of P2-NFMO-LV electrode.

| ***Parameters*** | ***1st Na+ extraction*** | | ***1st Na+ insertion*** | |
|---|---|---|---|---|
| | **3.69 V** | **4.15 V** | **2.42 V** | **1.50 V** |
| $R_{EEI}$ (Ω $mg^{-1}$) | 24.4 | 25.6 | 20.4 | 20.3 |
| $C_{EEI}$ (F $cm^{-2}s^{-(\alpha-1)}$) | $3.6e^{-7}$ | $3.9e^{-7}$ | $4.1e^{-7}$ | $3.8e^{-7}$ |
| $\alpha_{EEI}$ | 1.0 | 1.0 | 1.0 | 1.0 |
| $R_{CT}$ (Ω $mg^{-1}$) | 59.4 | 79.5 | 60.9 | 162.5 |
| $C_{DL}$ (F $cm^{-2}s^{-(\alpha-1)}$) | $1.1e^{-5}$ | $2.0e^{-5}$ | $1.3e^{-5}$ | $2.3e^{-5}$ |
| $\alpha_{DL}$ | 0.9 | 0.8 | 1.0 | 0.9 |
| $R_{elec}$ (Ω $mg^{-1}$) | 83.6 | 3205.7 | 244.7 | 10398.3 |
| $C_{elec}$ (F $cm^{-2}s^{-(\alpha-1)}$) | $1.4e^{-3}$ | $2.6e^{-3}$ | $1.2e^{-3}$ | $6.4e^{-4}$ |
| $\alpha_{elec}$ | 0.9 | 0.9 | 0.8 | 0.7 |
| $X^2$ | $6.5e^{-4}$ | $6.0e^{-4}$ | $7.2e^{-4}$ | $1.8e^{-3}$ |

**Table S3:** Values from the fits by Boukamp´s software of the impedance data at 3.71 V, 4.0 V, 2.44 V and 2.0 V of P2-NFMO-SV electrode.

| ***Parameters*** | ***$1^{st}$ $Na^+$ extraction*** | | ***$1^{st}$ $Na^+$ insertion*** | |
|---|---|---|---|---|
| | **3.71 V** | **4.00 V** | **2.44 V** | **2.00 V** |
| **$R_{EEI}$ (Ω $mg^{-1}$)** | 9.9 | 15.1 | 21.8 | 15.0 |
| **$C_{EEI}$ (F $cm^{-2}s^{-(\alpha-1)}$)** | $3.5e^{-6}$ | $4.9e^{-5}$ | $4.4e^{-5}$ | $3.4e^{-5}$ |
| **$\alpha_{EEI}$** | 0.7 | 0.7 | 0.7 | 0.8 |
| **$R_{CT}$ (Ω $mg^{-1}$)** | 33.3 | 19.6 | 48.4 | 121.3 |
| **$C_{DL}$ (F $cm^{-2}s^{-(\alpha-1)}$)** | $2.4e^{-4}$ | $8.9e^{-5}$ | $1.6e^{-4}$ | $1.4e^{-4}$ |
| **$\alpha_{DL}$** | 0.9 | 1.0 | 0.8 | 0.7 |
| **$R_{elec}$ (Ω $mg^{-1}$)** | 143.6 | 64.4 | 169.0 | 4056.9 |
| **$C_{elec}$ (F $cm^{-2}s^{-(\alpha-1)}$)** | $3.7e^{-3}$ | $5.6e^{-3}$ | $2.3e^{-3}$ | $1.8e^{-4}$ |
| **$\alpha_{elec}$** | 1.0 | 0.9 | 0.8 | 0.9 |
| **$X^2$** | $2.5e^{-3}$ | $4.7e^{-4}$ | $1.4e^{-4}$ | $9.5e^{-4}$ |
