# Supplementary material for: Role of the voltage window on the capacity retention of P2-Na2/3[Fe1/2Mn1/2]O2 cathode material for rechargeable sodium-ion batteries
Source: Commun Chem. 2022 Feb 1;5:11. doi: 10.1038/s42004-022-00628-0 (PMC9814619; doi:10.1038/s42004-022-00628-0)
Supplement: Supplementary file 4 — Description of Additional Supplementary Files [file 42004_2022_628_MOESM4_ESM.pdf]

Description of Additional Supplementary Files

File Name: Supplementary Data 1

Description: Atomic coordinates used in the electronic structure calculations are provided as .CIF file for the P2 phase.

File Name: Supplementary Data 2

Description: Atomic coordinates used in the electronic structure calculations are provided as .CIF file for the O2 phase
